# Patients with Knee Osteoarthritis Undergoing Total Knee Arthroplasty Have a Lower Risk of Subsequent Severe Cardiovascular Events: Propensity Score and Instrumental Variable Analysis

**DOI:** 10.1371/journal.pone.0127454

**Published:** 2015-05-26

**Authors:** Wen-Yan Lin, Ching-Chih Lee, Chia-Wen Hsu, Kuang-Yung Huang, Shaw-Ruey Lyu

**Affiliations:** 1 Department of Orthopedics, Dalin Tzu Chi Hospital, Buddhist Tzu Chi Medical Foundation, Chiayi, Taiwan; 2 Department of Otolaryngology, Dalin Tzu Chi Hospital, Buddhist Tzu Chi Medical Foundation, Chiayi, Taiwan; 3 Department of Education, Dalin Tzu Chi Hospital, Buddhist Tzu Chi Medical Foundation, Chiayi, Taiwan; 4 School of Medicine, Tzu Chi University, Hualian, Taiwan; 5 Center for Clinical Epidemiology and Biostatistics, Dalin Tzu Chi Hospital, Buddhist Tzu Chi Medical Foundation, Chiayi, Taiwan; 6 Department of Medical Research, Dalin Tzu Chi Hospital, Buddhist Tzu Chi Medical Foundation, Chia-Yi, Taiwan; 7 Division of Allergy, Immunology, and Rheumatology, Department of Internal Medicine, Dalin Tzu Chi Hospital, Buddhist Tzu Chi Medical Foundation, Chiayi, Taiwan; 8 Department of Life Science and Institute of Molecular Biology, National Chung Cheung University, Chiayi, Taiwan; 9 Joint Center, Dalin Tzu Chi Hospital, Buddhist Tzu Chi Medical Foundation, Chiayi, Taiwan; Oxford University, UNITED KINGDOM

## Abstract

**Objective:**

This population-based study investigated the subsequent cardiovascular risk of patients with knee osteoarthritis underwent total knee arthroplasty in Taiwan.

**Materials and methods:**

This was a population-based follow-up study of 22931 patients diagnosed with knee osteoarthritis between 2008 and 2011. Each patient was followed for 3 years or until death. Treatment was dichotomized into conservative treatment and TKA. The association between TKA and cardiovascular disease (CVD) events was analyzed using propensity score analysis and instrumental variable analysis and two-stage least-squares regression model.

**Results:**

Patients with knee osteoarthritis who underwent TKA had a lower 3-year cumulative risk of stroke and acute myocardial infarction (AMI). After adjusting for measured risk and confounding factors, propensity score showed a 0.56 fold (adjusted OR = 0.56; 95% CI, 0.51–0.61; *p*<0.001) risk for CVD in those with TKA. Use of instrumental variable analysis for adjusting measured and unmeasured factors and two-stage least squares regression model revealed that the average treatment effect of TKA was statistically associated with a decreased 7% risk of CVD events (95% CI, 0.2%–13.6%).

**Conclusion:**

Our study revealed that patients with knee osteoarthritis who underwent TKA had a lower risk of suffering from a future severe cardiovascular event. This benefit may be attributed to an improvement in physical activity, reduction of psychosocial stress, and/or a decreased use of NSAIDs as a result of having undergone TKA.

## Introduction

There is a high prevalence of symptomatic knee pain and radiographic knee osteoarthritis in the elderly [1, 2]. Total knee arthroplasty (TKA) is the primary forms of treatment for suitable candidates with advanced knee osteoarthritis (OA). The majority of patients who undergo TKA report a reduction in pain and improved gait, mobility, quality of life, and overall wellbeing following surgery, and this functional outcome has been emphasized for decades [3, 4]. These benefits are seen in all age groups, including in the extreme elderly[5]. As well as improvements in quality of life, increased mobility after knee arthroplasty may improve cardiovascular fitness[6]. Better cardiovascular fitness was associated with lower risk of coronary heart disease and cardiovascular disease[7].

More than 20% of the population of the United States aged 60 or older, are reported suffer from knee pain. The most common cause of knee pain is OA. Physical inactivity is associated with a higher prevalence of knee pain [1]. The American Academy of Orthopedic Surgeons (AAOS) Clinical Practice Guidelines “Treatment of Osteoarthritis of the Knee” strongly recommends the use of non-steroidal anti-inflammatory drugs (NSAIDs) for patients with symptomatic osteoarthritis of the knee[8]. However, NSAIDs have been proven to increase the risk of serious coronary heart disease when used in certain quantities [9]. Patients with knee osteoarthritis are at higher risk of death compared with the general population[10]. The severity of disability caused by knee OA or hip OA is associated with a significant increase in serious CVD events [11]. Bheeshma Ravi.[12] reported that patients who underwent a total joint arthroplasty during the exposure period were significantly less likely to experience a cardiovascular event than those who did not receive this treatment in a propensity score matched landmark analysis (hazard ratio, 0.56; 95% confidence interval, 0.43–0.74). However, overestimation of the treatment effect is frequently seen in observational studies, which just adjusted the observable bias, such as propensity score analysis.

For this study, we used Taiwan’s National Health Insurance Research Database (NHIRD) to investigate the subsequent risk of severe cardiovascular events in patients with knee OA who underwent TKA using propensity score and instrumental variable analysis in order to control the measured and unmeasured bias.

Instrumental variable methods have been used by social scientist and, more recently, by clinical researchers to overcome treatment selection bias [13, 14]. When an instrumental variable analysis works well, it provides high-quality evidence about a causal relationship.

An instrumental variable is a characteristic of the world that leads some people to be more likely to get the specific treatment we want to study but does not otherwise change those patients’ outcomes[15]. Intuitively, an instrumental variable analysis exploits a little bit of natural randomness in otherwise nonrandomized studies to create a situation that can be examined as if it were an RCT (randomized controlled trial).

## Materials and Methods

### Ethics statement

This study was approved by the Institutional Review Board of Buddhist Dalin Tzu Chi General Hospital in Taiwan. The Review Board did not require informed consent for this study because all personally identifiable information was removed from data collected from the National Health Insurance Research Database (NHIRD) prior to analysis.

### Database

The data for this study were collected from the NHIRD for the years 2008 to 2011. The NHIRD dataset is organized and managed by the Taiwan National Health Research Institutes. The original data was collected by the Taiwan National Health Insurance Program that was established in 1995. The program covers approximately 99% of all residents in Taiwan and has contracts with 97% of all medical providers in the nation[16]. In order to verify the accuracy of diagnosis and management, the Taiwan Bureau of National Health Insurance randomly reviews the charts of one per 100 ambulatory and one per 20 inpatients claims and also conducts patient interviews[17]. All patient data were reviewed retrospectively.

Our study cohort comprised patients with knee OA as identified by the International Classification of Diseases, Ninth Revision, Clinical Modification [ICD-9-CM] codes 715.16, 715.26, 715.36, and who had received treatment of knee OA for 4 or more times. Patients who received TKA were included. The patients were under 40 years old was excluded. In Taiwan, the TKA must being approved by Bureau of National Health Insurance preoperatively according to “Operational Guidelines Governing the Preapproval of Specific Diagnostic Items and Medications for National Health Insurance”[18]. The insurance data and radiography of knee are registered by orthopedist before operation. The TKA is approved if the radiographic OA meet the criteria: 1. knee joint space narrowing ≧ 50%, and ≧ 2 knee compartments involved for patient who was under 70 years old. 2. Knee joint space narrowing ≧ 50% for patient who was over 70 years old. All patients had poor response to conservative treatment for a minimum of 3 months. Therefore, all patients undergoing TKA in our database are Kellgren-Lawrence grade 3 or 4.

Due to an unequal distribution of patient characteristics during instrumental variable analysis, we only recruited patients who were treated or followed in northern/ central region for instrumental variable analysis. We followed the patients for 3 years, or until death, to observe the incidence of severe cardiovascular events (i.e., a stroke or AMI).

## Measurement*s*

The major independent variables of the study were the interaction effects of the knee OA patients who underwent TKA or conservative treatment. The enrolled patients were from the 2008 NHIRD data set.

Severe cardiovascular events—comprising AMI or stroke—and overall cumulative risk rate of each knee OA patient were determined by linking patient data from 2008 to 2011 with claims data. Patients were diagnosed as having a stroke according to claims data ICD-9M codes 430, 431, 432, 433, 434, 435, 436, 437, and 438. Patients were diagnosed as having suffered from an AMI according to claims data ICD-9M codes 410, 411, and 412. Patient characteristics were recorded, including age, sex, level of urbanization, socioeconomic status, geographic location, characteristics of hospital and comorbidity. Severity of disease was based on a modified Charlson Comorbidity Index Score (CCIS), which is widely accepted for risk adjustment in administrative claims data sets [19].

### Statistical analysis

All statistical analyses were performed using SPSS (version 15, SPSS Inc., Chicago, IL, USA). Pearson’s chi-square test was used for categorical variables such as age, sex, level of urbanization, geographic region, category of Charlson Comorbidity Index Score, and socioeconomic status. Continuous variables were analyzed using one-way ANOVA.

1. **Propensity score.** Propensity score stratification was applied to replace the wide host of observable confounding factors that may be present in an observational study with a variable of these factors. To derive the propensity score in this study, patient characteristics were entered into a logistic regression model predicting selection for TKA therapy. The characteristics included age, sex, Charlson Comorbidity Index score, socioeconomic status, level of urbanization, geographic area of residence, characteristics of hospital and treatment modality. The effect of 3-year CVD rate on TKA was analyzed within each quintile. The Mantel-Haenszel odds ratio was calculated in addition to performing the Cochran-Mantel-Haenszel χ^2^ test.
2. **Instrumental variable analysis.** Instrumental variable analysis was used to account for both measured and unmeasured confounding factors. The instrumental variable was estimated by first calculating the proportion of knee OA patients who received TKA in each hospital. Hospitals with one or more cases were included. High-use and low-use hospitals corresponded to the top and bottom quartiles of TKA utilization and were used as the binary instrumental variable for the binary treatment assignment. An instrumental variable must be associated with outcomes through its correlation with treatment status (TKA) and not through other variables. We verified this assumption by comparing the baseline characteristics, including age, sex, Charlson Comorbidity Index Score, socioeconomic status, level of urbanization and characteristics of hospital. Two-stage least-squares regression was used to estimate the effect of TKA using the instrumental variable.

## Results

A total of 22931 knee OA patients were included in this study (see Table 1). A total of 15363 patients received TKA in 2008. The remaining 7568 patients did not receive TKA. In comparison to the patients who had not undergone surgery, patients treated with TKA were female dominant, more likely to have a lower socioeconomic status, less comorbidity, live in a rural area, and have undergone treatment in a public hospital.

**Table 1:** Demographic characteristics for knee osteoarthritis patients (*n* = 22931).

Demographic characteristics	Without surgery7568(%)	TKA^+^15363(%)	*p* value
Age, years	70±12	70±8	0.797
Gender			<0.001
Female	4830(63.8)	11634(75.7)	
Male	2738(36.2)	3729(24.3)	
Charlson Comorbidity Index Score			<0.001
0	2863(37.8)	8264(53.8)	
≧1	4705(62.2)	7099(46.2)	
Socioeconomic status			<0.001
Low(≦NT $ 25000 or US $ 833)	7072(93.4)	14717(95.8)	
High(≧NT $ 25001 or US $ 833)	496(6.6)	646(4.2)	
Urbanization level			<0.001
Urban/ Suburban	4863(64.3)	8860(57.7)	
Rural	2705(35.7)	6503(42.3)	
Region			0.155
Northern/ Central	4451(58.8)	8884(57.8)	
Southern/Eastern	3117(41.2)	6479(42.2)	
Ownership			<0.001
Non for profit/ For profit	5252(69.4)	10155(66.1)	
Public	2316(30.6)	5208(33.9)	

+TKA, total knee arthroplasty.

At the end of the follow-up period, 2253 patients had CVD events, 1089 (7.1%) in those undergoing TKA and 1164 (15.4%) in those not using it (see Table 2). Patients using TKA exhibited a decreased risk of CVD events (7.1%: 15.4%, *p*<0.001).

**Table 2:** Severe vascular events in study population (*n* = 22931).

	Without surgery n = 7568(%)	TKA^+^ n = 15363(%)	*p* value
Acute myocardial infarction	264(3.5)	261(1.7)	<0.001
Stroke	966(12.8)	865(5.6)	<0.001
Acute myocardial infarction or stroke	1164(15.4)	1089(7.1)	<0.001

+TKA, total knee arthroplasty.

**Fig 1:**
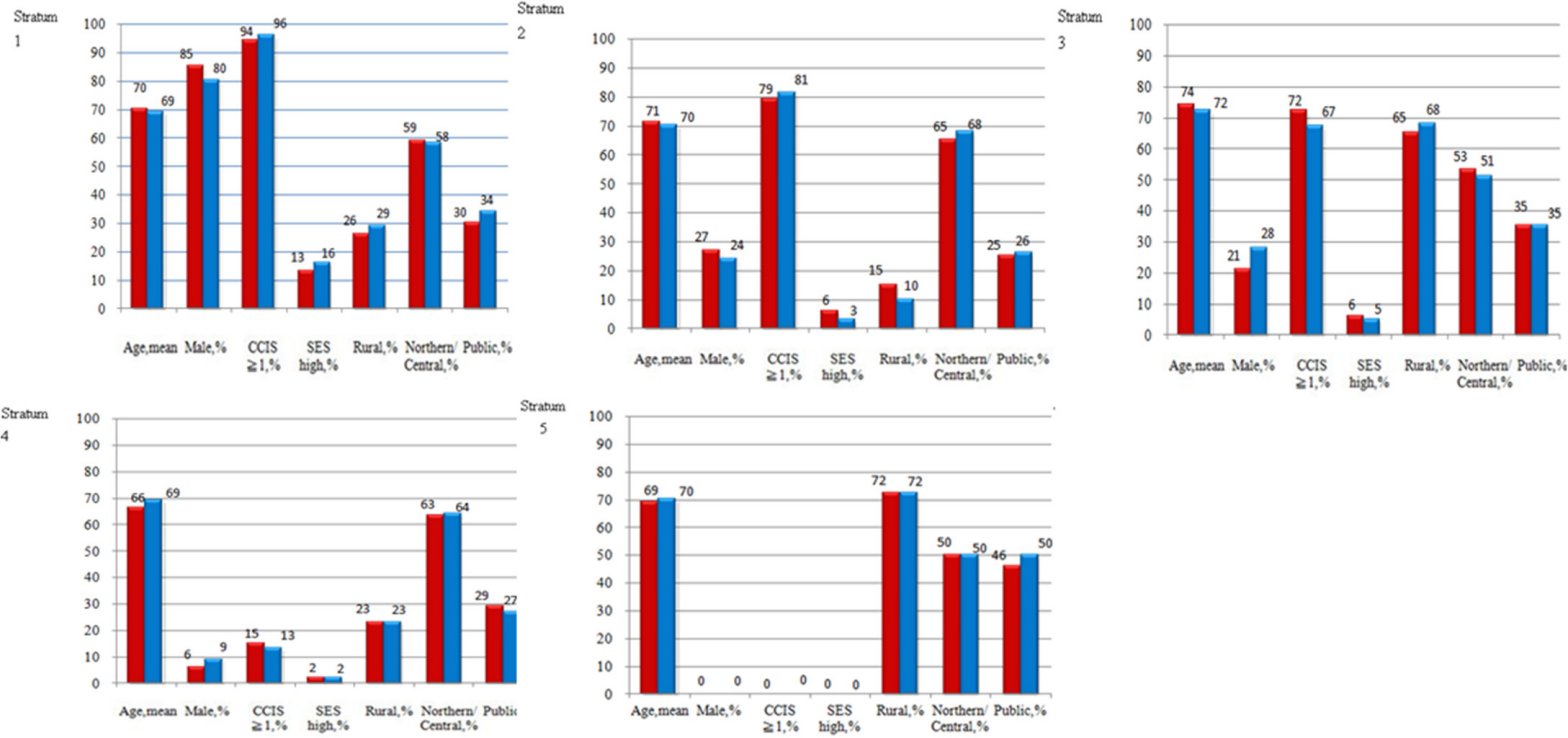
Distribution of explanatory variables between patients receiving TKA and those not receiving TKA for propensity score quintiles ranging from stratum 1 (least likely to receive TKA) to stratum 5 (most likely to receive TKA).

Propensity score stratification was applied, patients receiving TKA and those not receiving TKA for propensity score quintiles ranging from stratum 1 (least likely to receive TKA) to stratum 5 (most likely to receive TKA). The covariates entered into the propensity score were age, sex, Charlson Comorbidity Index score, socioeconomic status, level of urbanization, geographic area of residence, and treatment in public hospital (Fig 1).

The Table 3 shows the CVD event rates for “with TKA” and “without TKA” groups after stratification. The *p*-value for Cochran-Mantel-Haenszel statistics comparing CVD rates for TKA therapy and without surgery, controlling for propensity scores, was <0.001. Patients treated with TKA had lower CVD rates. The adjusted CVD rates for patients treated with TKA were lower than patients without TKA (adjusted OR 0.56; 95% CI, 0.51–0.61; *p* = 0.001).

**Table 3:** Three-years cumulative risk of severe vascular events among the patients by treatment modality.(*n* = 22931).

Stratum	No.		%of Stratum		Risk(%)		
	Without surgery	TKA^+^	Without surgery	TKA	Without surgery	TKA	*p* value
1	2207	2380	0.48	0.52	23.9	16.1	<0.001
2	1706	2880	0.37	0.63	18.0	10.3	<0.001
3	1500	3085	0.33	0.67	18.7	10.6	<0.001
4	1317	3270	0.29	0.71	3.6	2.3	0.013
5	838	3748	0.18	0.82	0.2	0.2	0.994
Total	7568	15363			15.4	7.1	<0.001

+TKA, total knee arthroplasty.

Stratum 1 had the strongest propensity for not receiving TKA; stratum 5, for receiving TKA. Conchran-Mantel-Haenszel statistics; adjusted odds ratio = 0.56, 95% confidence interval = 0.51–0.61.

**Fig 2:**
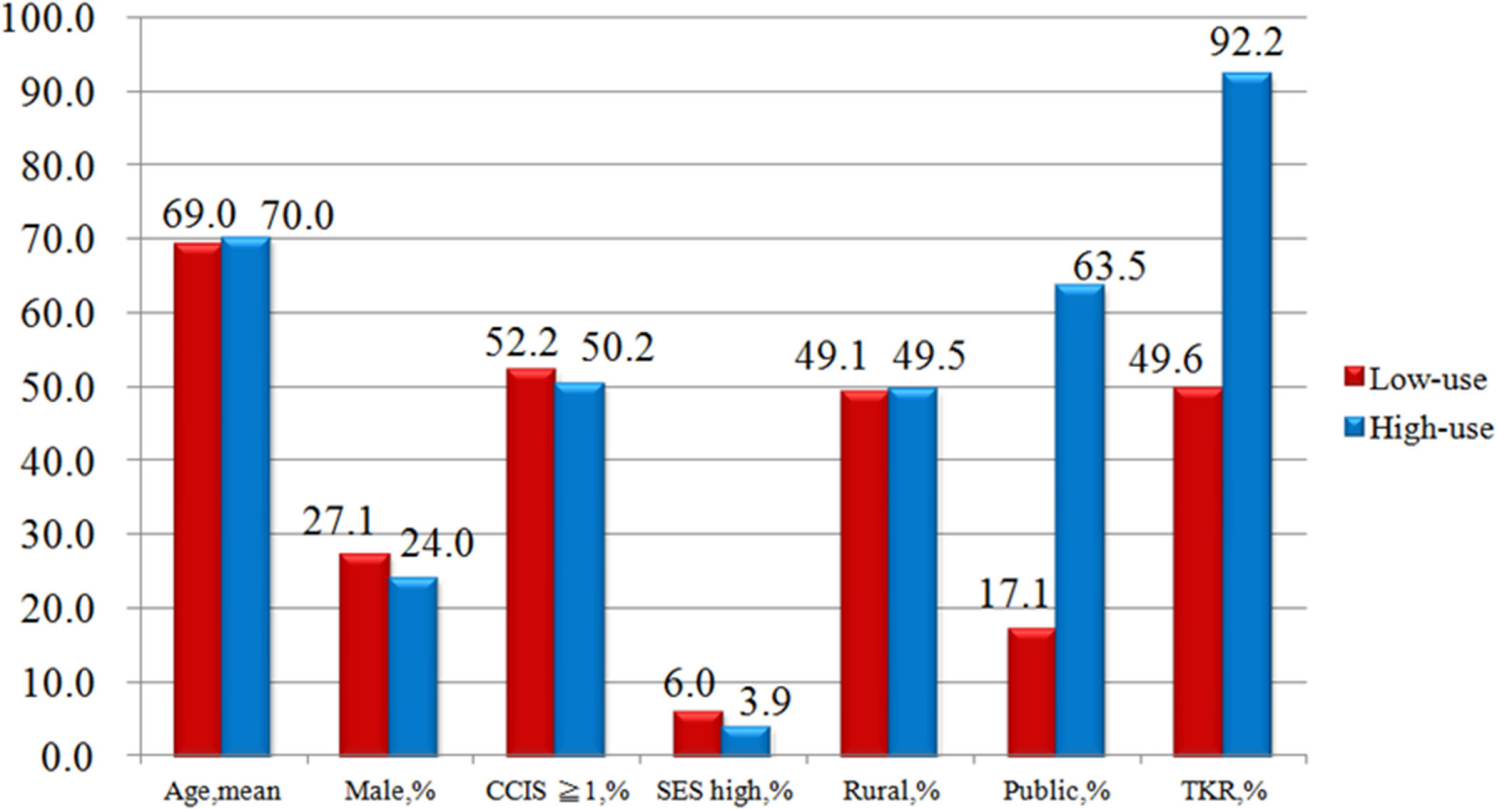
Distribution of explanatory variables between patients in high-use and low-use TKA hospitals.

**Fig 3:**
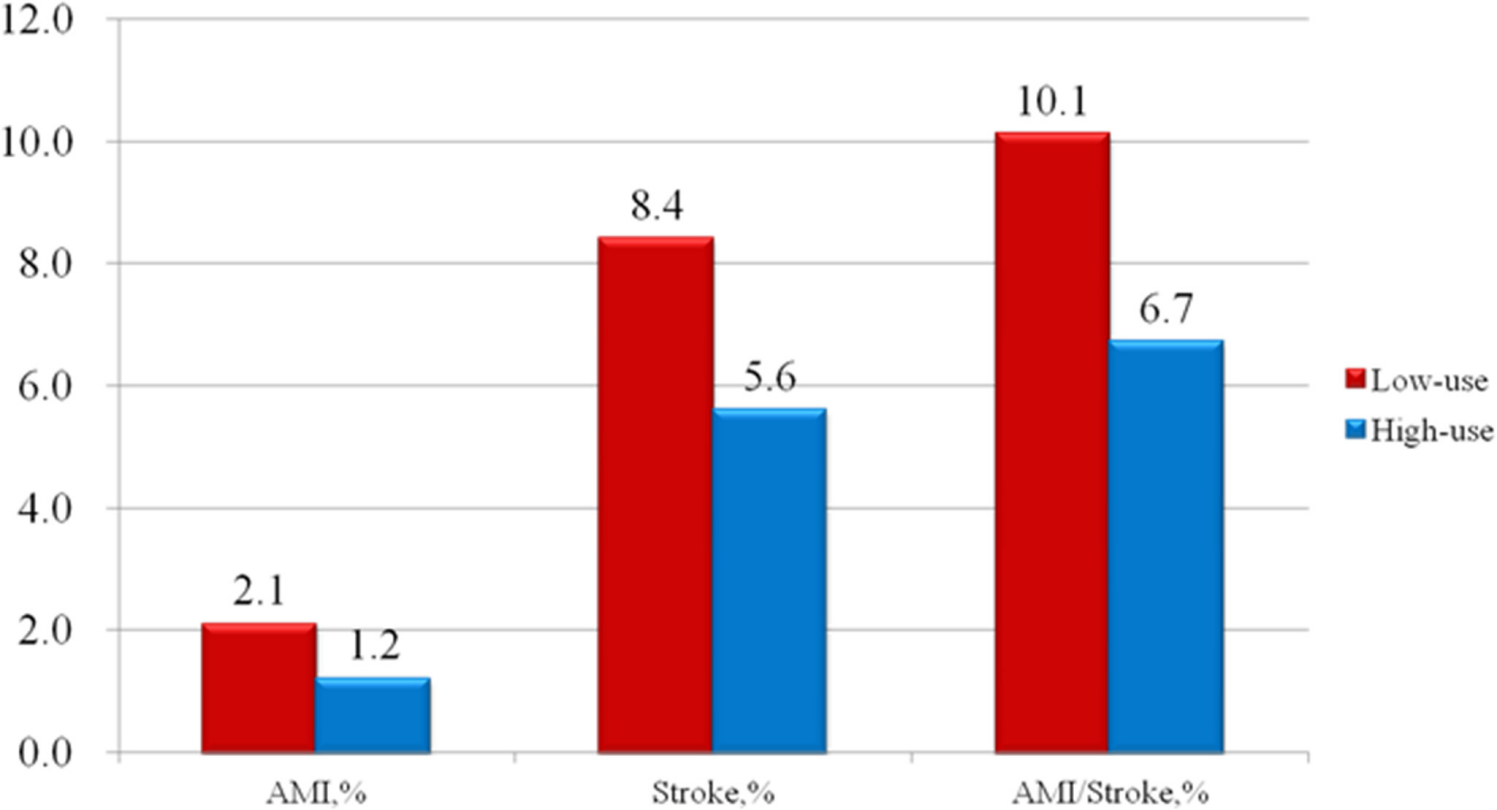
Distribution of explanatory variables between patients in high-use and low-use TKA hospitals and severe cardiovascular events.

Propensity score analysis is unable to adjust for unmeasured confounders and selection biases. For example, higher-risk patients may be preferentially selected for conservative treatment, thus producing apparently adverse outcomes for this group. Among the IVA, there were no statistical differences between the patients’ characteristics in high-use (*n* = 643) and low-use (*n* = 898) hospitals (see Table 4). TKA utilization varied widely across healthcare providers (49.6%–92.2%) (Fig 2). There are 10.1% patients in low use TKA group developed CVD during the follow up, comparatively there are 6.7% patients in high use TKA group developed CVD (Fig 3). Using two-stage least-squares analysis (see Table 5), TKA was statistically associated with a reduction of 7% in CVD events (95% CI, 0.2%–13.6%; *p* = 0.048).

**Table 4:** Characteristics of knee osteoarthritis patients in high-total knee arthroplasty and low- total knee arthroplasty use hospitals (*n* = 1541).

	Low-use *n* = 898(%)	High-use *n* = 643(%)	*p* value
Age, years	69±10	70±8	0.588
Male gender	243(27.1)	154(24.0)	0.169
Charlson Comorbidity Index Score≧1	469(52.2)	323(50.2)	0.440
High socioeconomic status (NT≧ $ 25001 or US $ 833)	54(6.0)	25(3.9)	0.062
Urbanization level: Rural	441(49.1)	318(49.5)	0.893
Ownership: Public	154(17.1)	408(63.5)	<0.001
Total knee arthroplasty	445(49.6)	593(92.2)	<0.001

Parenthesis is percentage of patients in high-use or low-use hospitals

**Table 5:** Marginal effect of different treatment on severe vascular events using instrumental variable analysis for three years follow-up (*n* = 1541).

Variables	Odds ratio	95% CI*	*p* value
Total knee arthroplasty	0.970	(0.942–0.999)	0.048
Age, years	1.002	(1.000–1.004)	0.003
Male gender	1.020	(0.989–1.053)	0.212
Charlson Comorbidity Index Score≧1	1.164	(1.133–1.197)	<0.001
High socioeconomic status (NT≧ $ 25001 or US $ 833)	0.967	(0.908–1.029)	0.296
Urbanization level: Rural	1.009	(0.982–1.037)	0.518
Ownership: Public	0.965	(0.939–0.991)	0.013

* two stage least square results: (1–0.97) / (0.922–0.496) = 0.07

HR: 93%, 95% CI; 0.2%–13.6%; *p* = 0.048

## Discussion

Our data showed that knee OA patients who underwent TKA have a lower risk of subsequent severe cardiovascular events than patients who did not undergo TKA. The cumulative risk rate was more favorable among patients who underwent TKA after adjusting for the patients’ sex, age, level of urbanization, geographic region, Charlson Comorbidity Index Score using propensity score analysis, and instrumental variable analysis. Orthopedists should recognize the potential benefit to cardiovascular health that TKA provides to patients with knee OA.

The strength of our study is that it was a population-based observational study and had an adequate number of patients to avoid minor confounding factors. Taiwan’s NHI program has covered approximately 99% of residents since 1995, and the validity of the dataset has been verified.

We observed that knee OA patients who underwent TKA had independent effects on incidence of subsequent severe cardiovascular events. Propensity score analysis simulated the randomization process in an attempt to eliminate selection bias on observables and revealed about a 44% decreased risk of CVD events for patients with TKA. Using IVA to control both measured and unmeasured confounding factors, there was a statistically significant difference (hazard ratio = 0.93, 7% decreased risk) between the CVD rate and TKA. Severity of comorbidities, BMI, employment, physical activity along with patient preferences was difficult to capture correctly from the dataset. Referral selection may depend on the interactions between the comorbidities and patient preferences. All these unmeasured factors would have produced a significant bias using traditional approaches.

In our study, the risk of subsequent severe cardiovascular events was decreased among patients who underwent TKA within 3 years of follow up. However, using IVA controlling both the measured and unmeasured bias, the protective effect of surgical intervention for knee OA is about 7%, which was different from the results of Ravi Bheeshma *et al*.[12], who reported a significant (40%) reduction in subsequent risk of serious cardiovascular events in patients who underwent total joint arthroplasty based on a propensity score matched landmark analysis. The previous study using propensity score to adjust observable bias may overestimate the protective effect of joint arthroplasty.

Total knee arthroplasty has several potential benefits to prevent cardiovascular disease. It can improve the capacity for physical activity in most patients. Physical activity can improve and maintain health in older adult [20]. The strongest evidence to date was obtained in a 2 year follow up study which was later extended to 5 year and 10 year follow-ups, all of them showed similarly positive results [21]. Exercise and physical activity during leisure time is associated with a significantly reduced incidence rate of coronary disease [22, 23], hypertension, and diabetes [24, 25].

Another potential cardiovascular benefit of TKA is a reduction in psychosocial stress. A sizeable percentage of older adults with knee OA may experience latent symptoms of depression that range from mild to severe [26]. Those with lower self-efficacy beliefs for carrying out self-management activities are more likely to present with more frequent depressive symptoms than those with a high degree of self-efficacy. Observational studies have indicated that psychosocial factors, such as depression and anxiety, lack of social support, social isolation, and stressful conditions independently influence the occurrence of major risk factors and the course of coronary heart disease [27].

One limitation of this study is that the diagnosis of knee OA as well as comorbidities (such as hypertension[28], diabetes[29], and chronic renal failure) was collected from ICD-9-CM codes on National Health Insurance claims. However, the NHI Bureau in Taiwan regularly conducts randomized reviews of the charts and interviews patients to spot-check the accuracy of diagnoses, and the NHIRD appears to be a valid resource for population research [30]. Secondly, hip OA, hip osteonecrosis, and ankle OA are also leading causes of disability among the elderly, but these conditions were not included in our study. It is possible that these conditions could result in a negative physical activity outcome in patients who undergo TKA. Nevertheless, we hypothesize that activity resumption as a secondary measure with arthroplasty provides possible benefits in preventing subsequent cardiovascular disease. Another limitation is that we were unable to acquire any information concerning preoperative and postoperative physical activity, psychosocial stress, or use of NSAIDs during the period of study, which are potential factors in the prevention of cardiovascular events and unmeasured biases, such as disease severity [31]. However, IVA could eliminate the selection biases from the unmeasured factors.

## Conclusion

Our study revealed that patients with knee OA who underwent TKA have a lower risk of subsequent severe cardiovascular events. These results provide insight into the cardioprotective effect of TKA. The effect of TKA involves not only an improved quality of life and increased physical activity, but also highlights the cardiovascular protective benefit of TKA. Our findings should be confirmed in a larger study, which would help establish a more direct relationship between the improvement in physical activity, reduction of psychosocial stress, decreased use of NSAIDs, and a subsequently reduced risk of cardiovascular disease in patients who undergo TKA.
